# Supplementary material for: Ethanol Induces Extracellular Vesicle Secretion by Altering Lipid Metabolism through the Mitochondria-Associated ER Membranes and Sphingomyelinases
Source: Int J Mol Sci. 2021 Aug 5;22(16):8438. doi: 10.3390/ijms22168438 (PMC8395151; doi:10.3390/ijms22168438)
Supplement: Supplementary file 1 [file ijms-22-08438-s001.zip › ijms-1298239-supplementary.pdf]

SUPPLEMENTARY MATERIAL

Figure S1. Uncropped blots of p-p65, p65, p-ERK, ERK, p-p38, p38, GAPDH, NLRP3, TLR4, IL-1R, HSP70 and CD81.

A)

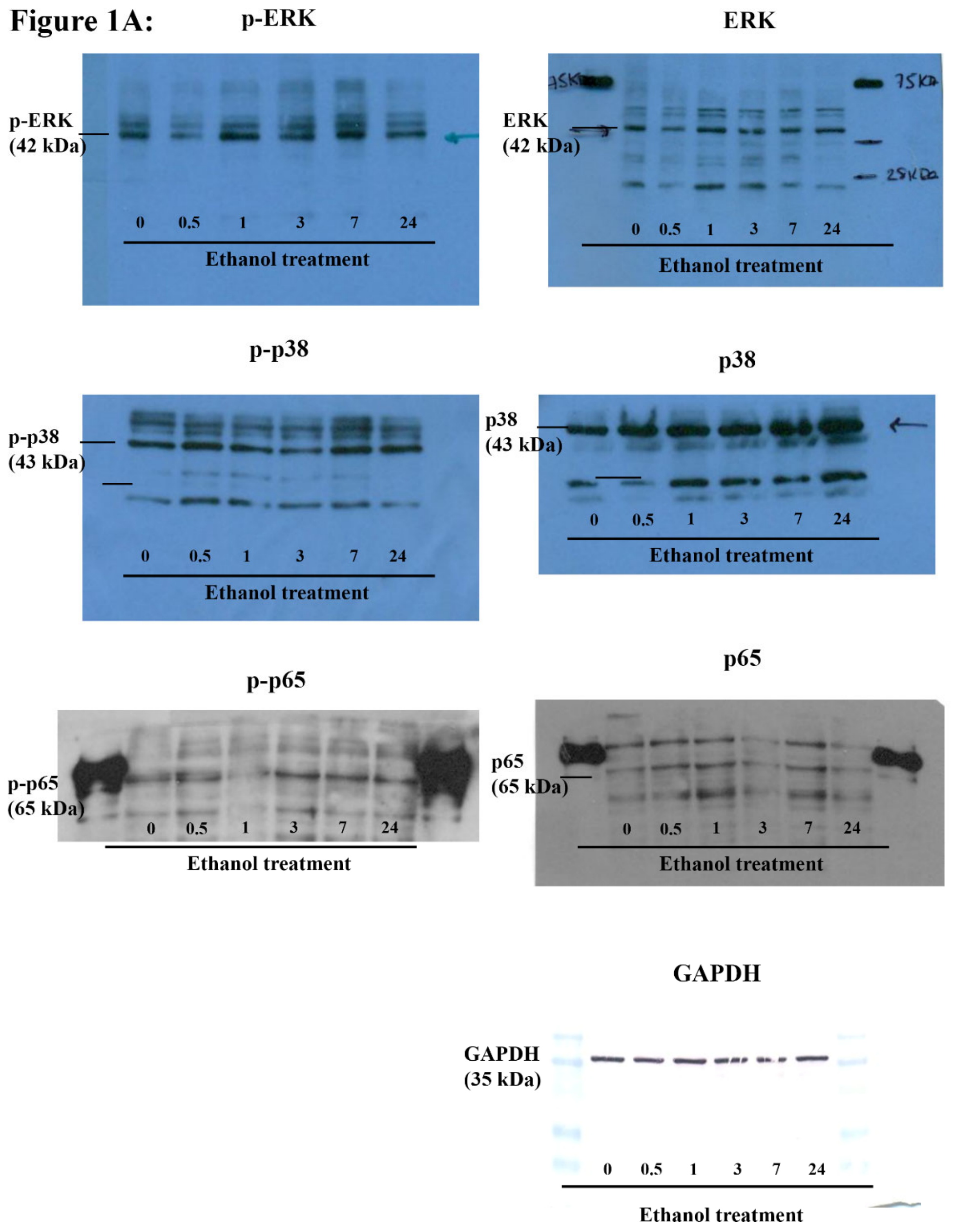

B)

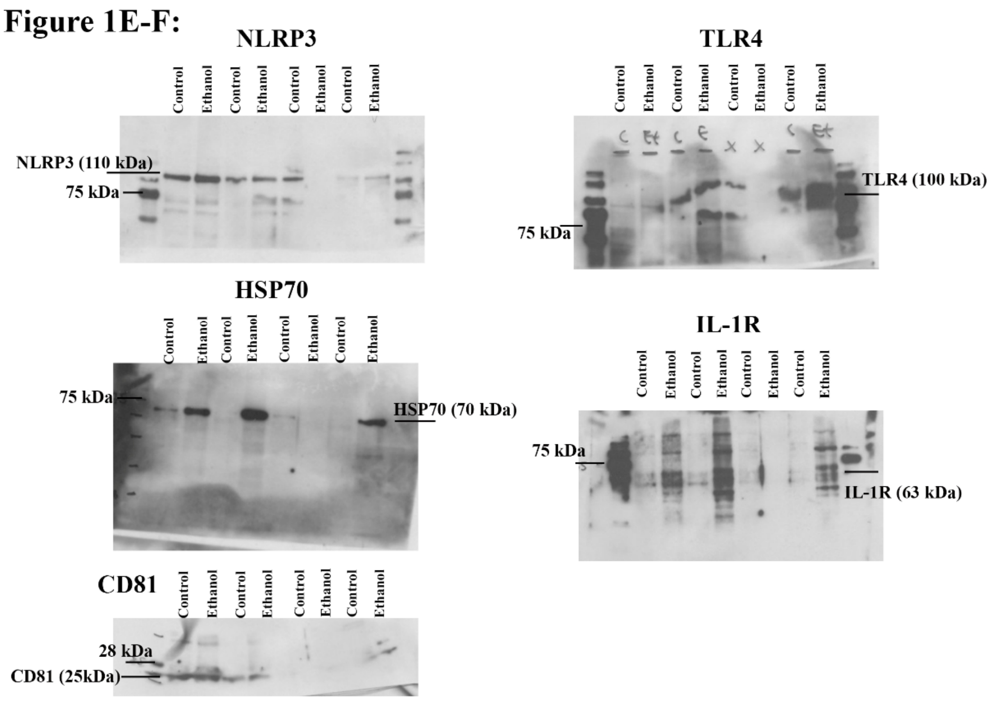

Figure S2: Panoramic images of microscopic studies. A) Uncropped confocal images of the BV2 phagocytic study, one for each experimental condition. B) Transmission electron microscopy images of EVs isolated from the supernatant of BV2 cells. Areas selected in red squares were used in Figures 1B and 1C. C) The individual channels for the images in Figure 1B are shown below.

A)

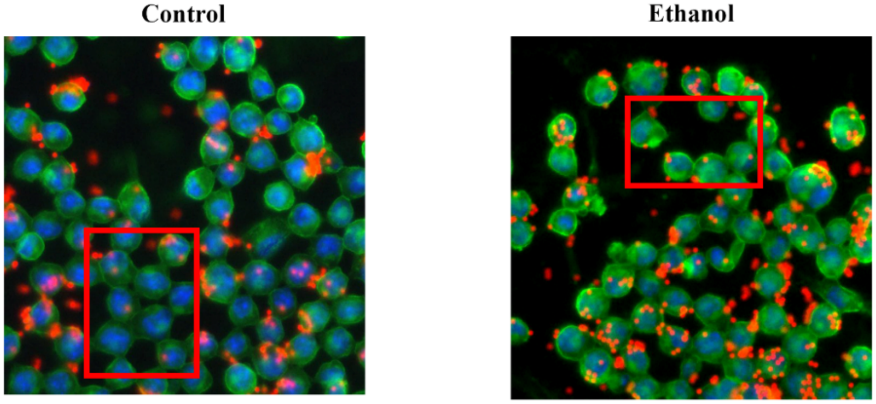

B)

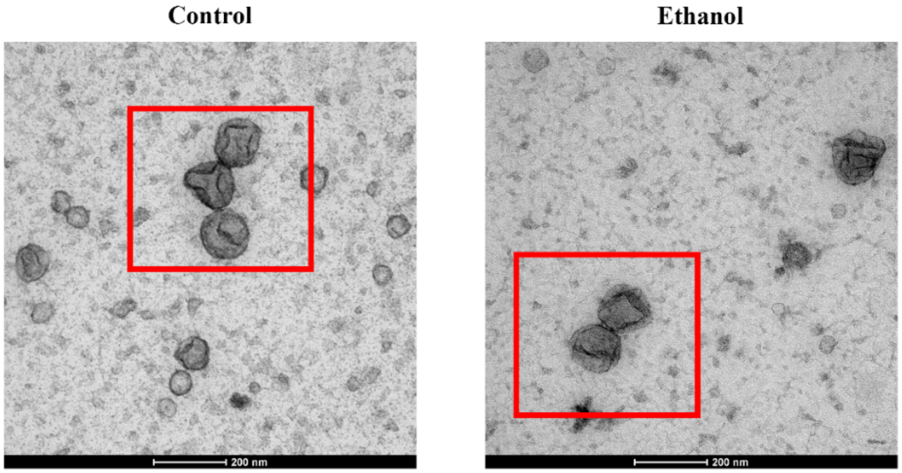

C)

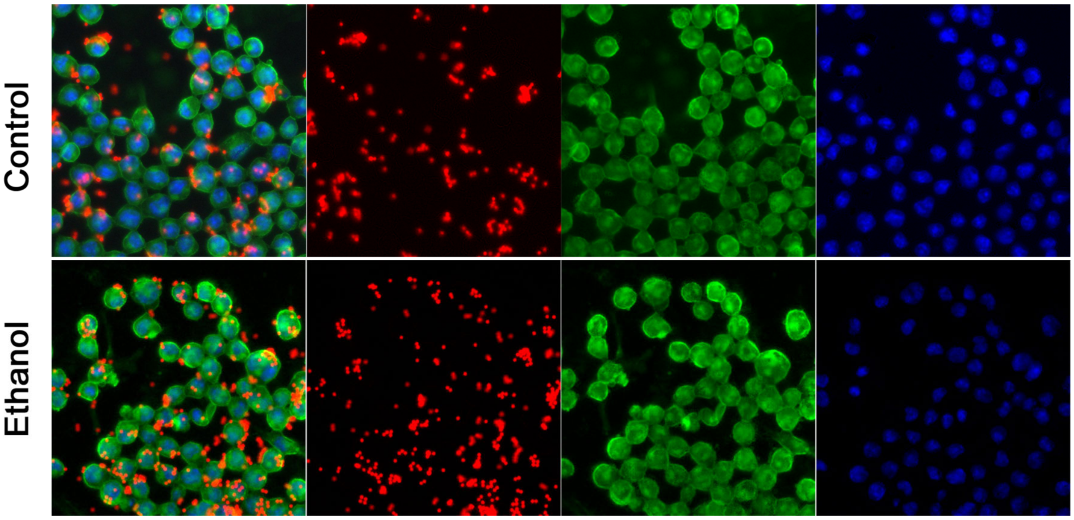

Figure S3: A graphical representation of the size range distribution of the EVs isolated from the supernatant of BV2 cells for each experimental condition described in Figure 4B.

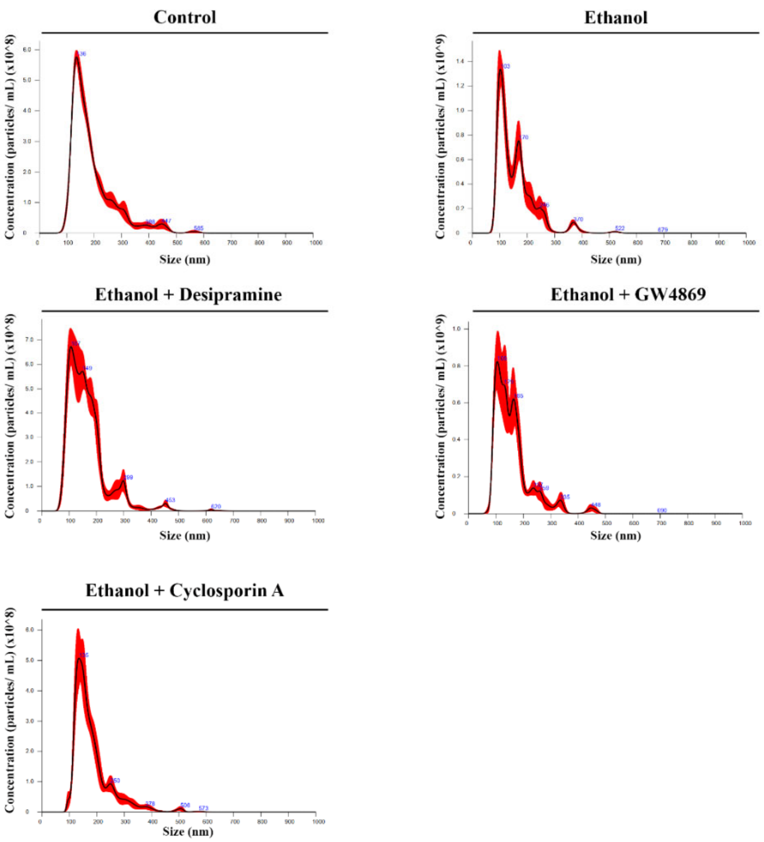

Table S1. Nucleotide sequences of the primers used for RT-PCR of micro-RNAs.

MicroRNA	Chromosome Location	Mature Primer sequences (5' to 3')
Mir-146a-5p	Chr.5: 160485352 - 160485450 [+] on Build GRCh38	UGAGAACUGAAUUCCAUGGGUU
Let-7b	Chr.22: 46113686 - 46113768 [+] on Build GRCh38	UGAGGUAGUAGGUUGUGUGGUU
Mir-451a	Chr.17: 28861369 - 28861440 [-] on Build GRCh38	AAACCGUUACCAUUACUGAGUU

Table S2. Statistical tests and results obtained for each experimental procedure.

Figures	Subfigures	Sample	p-value	Statistics
Figure 1	A	p-p65, Control vs. 30'	0,5984	Kruskal Wallis
		p-p65, Control vs. 1h	0,1201	
		p-p65, Control vs. 3h	0,0002	
		p-p65, Control vs. 7h	0,0005	
		p-p65, Control vs. 24h	0,0016	
		p-ERK, Control vs. 30'	0,0015	
		p-ERK, Control vs. 1h	0,0002	
		p-ERK, Control vs. 3h	0,0183	
		p-ERK, Control vs. 7h	0,0468	
		p-ERK, Control vs. 24h	0,0171	
		p-p38, Control vs. 30'	0,267	
		p-p38, Control vs. 1h	0,0143	
		p-p38, Control vs. 3h	0,0007	
		p-p38, Control vs. 7h	0,2466	
		p-p38, Control vs. 24h	0,0338	
	B	Control vs Etanol	0,0231	t-test
	D	Control vs Etanol (0-100 nm)	>0,9999	One Way ANOVA
		Control vs Etanol (100-200 nm)	0,0001	
		Control vs Etanol (200-300 nm)	0,9984	
		Control vs Etanol (300-400 nm)	0,9946	
	E	HSP70 Control vs Etanol	0,0211	t-test
		CD81 Control vs Etanol	0,0002	Mann-Whitney
	F	TLR4 Control vs Etanol	0,0433	t-test
		NLRP3 Control vs Etanol	0,0428	
		IL1R Control vs Etanol	0,1522	
	G	mir-146a Control vs Etanol	0,0253	
		mir-21 Control vs Etanol	0,0286	
		let-7b Control vs Etanol	0,0286	
Figure 2	A	Control vs Ethanol 50 mM 2 hours	0,6767	Two Way ANOVA
		Control vs Ethanol 50 mM 4 hours	0,0068	
		Control vs Ethanol 100 mM 2 hours	0,1304	
		Control vs Ethanol 100 mM 4 hours	0,1297	
	B	Control vs Ethanol 50 mM 2 hours	0,7258	
		Control vs Ethanol 50 mM 4 hours	0,0325	
		Control vs Ethanol 100 mM 2 hours	0,7709	
		Control vs Ethanol 100 mM 4 hours	0,4625	
	C	Control vs Ethanol 50 mM	0,021	
		Control vs Ethanol 100 mM	0,0273	

Figures	Subfigures	Sample	p-value	
Figure 3	A	PtdSer 0 vs 10	0,0459	One Way ANOVA
		PtdSer 0 vs 50	0,0008	
		PtdSer 0 vs 100	<0,0001	
		PtdEtn 0 vs 10	>0,9999	Kruskal Wallis
		PtdEtn 0 vs 50	0,0204	
		PtdEtn 0 vs 100	0,0218	
		PtdChol 0 vs 10	>0,9999	
		PtdChol 0 vs 50	0,605	
		PtdChol 0 vs 100	>0,9999	
	B	PtdSer Control vs Ethanol 50 mM 2 hours	> 0.9999	Two way ANOVA
		PtdSer Control vs Ethanol 50 mM 4 hours	0,095	
		PtdSer Control vs Ethanol 100 mM 2 hours	0,0221	
		PtdSer Control vs Ethanol 100 mM 4 hours	0,0086	
		PtdEtn Control vs Ethanol 50 mM 2 hours	> 0.9999	
		PtdEtn Control vs Ethanol 50 mM 4 hours	0,0997	
		PtdEtn Control vs Ethanol 100 mM 2 hours	0,0227	
		PtdEtn Control vs Ethanol 100 mM 4 hours	0,0019	
		PtdChol Control vs Ethanol 50 mM 2 hours	> 0.9999	
		PtdChol Control vs Ethanol 50 mM 4 hours	0,0492	
		PtdChol Control vs Ethanol 100 mM 2 hours	0,0169	
		PtdChol Control vs Ethanol 100 mM 4 hours	0,1	
	C	PtdSer Control vs Ethanol 50 mM 2 hours	> 0.9999	
		PtdSer Control vs Ethanol 50 mM 4 hours	0,3115	
		PtdSer Control vs Ethanol 100 mM 2 hours	> 0.9999	
		PtdSer Control vs Ethanol 100 mM 4 hours	0,0311	
		PtdEtn Control vs Ethanol 50 mM 2 hours	> 0.9999	
		PtdEtn Control vs Ethanol 50 mM 4 hours	0,3402	
		PtdEtn Control vs Ethanol 100 mM 2 hours	> 0.9999	
		PtdEtn Control vs Ethanol 100 mM 4 hours	0,0149	
		PtdChol Control vs Ethanol 50 mM 2 hours	> 0.9999	
		PtdChol Control vs Ethanol 50 mM 4 hours	0,5805	
		PtdChol Control vs Ethanol 100 mM 2 hours	> 0.9999	
		PtdChol Control vs Ethanol 100 mM 4 hours	0,033	
Figure 4	A	PtdSer Control Ethanol 0 vs 50 mM	0,0218	
		PtdSer Control Ethanol 0 vs 100 mM	0,0024	
		PtdSer Desipramine Ethanol 0 vs 50 mM	0,6833	
		PtdSer Desipramine Ethanol 0 vs 100 mM	0,0273	
		PtdSer Gw4869 Ethanol 0 vs 50 mM	>0,9999	
		PtdSer GW4869 Ethanol 0 vs 100 mM	>0,9999	
		PtdSer Cyclosporin A Ethanol 0 vs 50 mM	0,0657	
		PtdSer Cyclosporin A Ethanol 0 vs 100 mM	0,0786	
		PtdEtn Control Ethanol 0 vs 50 mM	0,0008	
		PtdEtn Control Ethanol 0 vs 100 mM	0,0008	
		PtdEtn Desipramine Ethanol 0 vs 50 mM	0,0026	

		PtdEtn Desipramine Ethanol 0 vs 100 mM	0,0016	
		PtdEtn Gw4869 Ethanol 0 vs 50 mM	0,3404	
		PtdEtn GW4869 Ethanol 0 vs 100 mM	>0,9999	
		PtdEtn Cyclosporin A Ethanol 0 vs 50 mM	0,0774	
		PtdEtn Cyclosporin A Ethanol 0 vs 100 mM	0,0217	
		PtdChol Control Ethanol 0 vs 50 mM	0,0001	
		PtdChol Control Ethanol 0 vs 100 mM	<0,0001	
		PtdChol Desipramine Ethanol 0 vs 50 mM	0,1625	
		PtdChol Desipramine Ethanol 0 vs 100 mM	0,3154	
		PtdChol Gw4869 Ethanol 0 vs 50 mM	0,4046	
		PtdChol GW4869 Ethanol 0 vs 100 mM	0,0211	
		PtdChol Cyclosporin A Ethanol 0 vs 50 mM	>0,9999	
		PtdChol Cyclosporin A Ethanol 0 vs 100 mM	>0,9999	
	B	Control vs Ethanol	0,0475	Kruskal Wallis
		Control vs Desipramine	>0,9999	
		Control vs GW4869	>0,9999	
		Control vs Cyclosporin A	>0,9999	

Table S3: Normality test by Shapiro-Wilk and IQR of every data group.

Figures	Subfigures	Sample	Passed Normality test?	IQR
Figure 1	B	Control	Yes (0,069)	0,73
		Ethanol	Yes (0,389)	0,89
	D	Control 0-100	Yes (0,651)	485093141
		Ethanol 0-100	Yes (0,943)	503832241
		Control 100-200	Yes (0,184)	1565052383
		Ethanol 100-200	Yes (0,193)	2197132398
		Control 200-300	Yes (0,403)	523829410
		Ethanol 200-300	Yes (0,378)	504847600
		Control 300-400	Yes (0,906)	143766349
		Ethanol 300-400	Yes (0,365)	77945673
	E	HSP70 Control	Yes (0,472)	1,561
		HSP70 Ethanol	Yes (0,308)	4,7
		CD81 Control	Yes (0,715)	3341
		CD81 Ethanol	No (0,0003)	40550
	F	TLR4 Control	Yes (0,616)	0,7959
		TLR4 Ethanol	Yes (0,165)	0,84
		NLRP3 Control	Yes (0,218)	0,6919
		NLRP3 Ethanol	Yes (0,908)	0,46
		IL1R Control	Yes (0,501)	1,118
		IL1R Ethanol	Yes (0,372)	1,6
	G	mir-146a Control	Yes (0,390)	2,916E-08
		mir-146a Ethanol	Yes (0,197)	2,314E-07
		mir-21 Control	Yes (0,226)	1,785E-08

		mir-21 Ethanol	Yes (0,366)	2,301E-07
		let-7b Control	Yes (0,325)	6,213E-10
		let-7b Ethanol	Yes (0,963)	1,177E-08
Figure 2	A	Cholesterol Uptake Control 2h	Yes (0,995)	478
		Cholesterol Uptake Control 4h	Yes (0,147)	3645
		Cholesterol Uptake Ethanol 50 mM 2h	Yes (0,074)	3801
		Cholesterol Uptake Ethanol 50 mM 4h	No (0,034)	10296
		Cholesterol Uptake Ethanol 100 mM 2h	Yes (0,799)	2930
		Cholesterol Uptake Ethanol 100 mM 4h	Yes (0,071)	8740
	B	Cholesterol Esterification Control 2h	Yes (0,999)	14
		Cholesterol Esterification Control 4h	Yes (0,463)	12
		Cholesterol Esterification Ethanol 50 mM 2h	Yes (0,999)	20
		Cholesterol Esterification Ethanol 50 mM 4h	Yes (0,482)	27
		Cholesterol Esterification Ethanol 100 mM 2h	Yes (0,317)	17
		Cholesterol Esterification Ethanol 100 mM 4h	Yes (0,565)	10
	C	Smase Control	Yes (0,869)	119
		Smase Ethanol 50 mM	Yes (0,935)	82
		Smase Ethanol 100 mM	Yes (0,977)	150
Figure 3	A	PtdSer 0 mM	Yes (0,189)	0,4087
		PtdSer 10 mM	Yes (0,986)	0,5879
		PtdSer 50 mM	Yes (0,210)	0,8202
		PtdSer 100mM	Yes (0,248)	1,347
		PtdEtn 0 mM	No (0,026)	0,4015
		PtdEtn 10 mM	Yes (0,762)	0,5445
		PtdEtn 50 mM	Yes (0,257)	0,8306
		PtdEtn 100mM	Yes (0,880)	1,147
		PtdChol 0 mM	No (0,045)	0,5916
		PtdChol 10 mM	Yes (0,801)	1,314
		PtdChol 50 mM	Yes (0,077)	3,084
		PtdChol 100mM	No (0,011)	3,415
	B	Acute PtdSer Control 2h	Yes (0,373)	1,083
		Acute PtdSer Control 4h	Yes (0,891)	2,157
		Acute PtdSer Ethanol 50 mM 2h	Yes (0,373)	1,324
		Acute PtdSer Ethanol 50 mM 4h	Yes (0,167)	4,452
		Acute PtdSer Ethanol 100 mM 2h	Yes (0,124)	2,794
		Acute PtdSer Ethanol 100 mM 4h	Yes (0,924)	4,763
		Acute PtdEtn Control 2h	Yes (0,986)	1,004
		Acute PtdEtn Control 4h	Yes (0,509)	1,153
		Acute PtdEtn Ethanol 50 mM 2h	Yes (0,580)	1,659
		Acute PtdEtn Ethanol 50 mM 4h	Yes (0,391)	2,837
		Acute PtdEtn Ethanol 100 mM 2h	Yes (0,824)	2,821
		Acute PtdEtn Ethanol 100 mM 4h	Yes (0,159)	7,685
		Acute PtdChol Control 2h	Yes (0,365)	1,046
		Acute PtdChol Control 4h	Yes (0,730)	4,032

		Acute PtdChol Ethanol 50 mM 2h	Yes (0,342)	0,5373
		Acute PtdChol Ethanol 50 mM 4h	Yes (0,345)	6,383
		Acute PtdChol Ethanol 100 mM 2h	Yes (0,542)	4,984
		Acute PtdChol Ethanol 100 mM 4h	Yes (0,464)	5,237
	C	Sustained PtdSer Control 2h	Yes (0,992)	1,394
		Sustained PtdSer Control 4h	Yes (0,132)	1,521
		Sustained PtdSer Ethanol 50 mM 2h	Yes (0,874)	1,295
		Sustained PtdSer Ethanol 50 mM 4h	No (0,015)	2,459
		Sustained PtdSer Ethanol 100 mM 2h	Yes (0,989)	1,421
		Sustained PtdSer Ethanol 100 mM 4h	Yes (0,235)	3,01
		Sustained PtdEtn Control 2h	Yes (0,844)	0,8657
		Sustained PtdEtn Control 4h	Yes (0,065)	1,234
		Sustained PtdEtn Ethanol 50 mM 2h	Yes (0,946)	0,8879
		Sustained PtdEtn Ethanol 50 mM 4h	Yes (0,074)	3,104
		Sustained PtdEtn Ethanol 100 mM 2h	Yes (0,310)	0,8194
		Sustained PtdEtn Ethanol 100 mM 4h	Yes (0,238)	3,649
		Sustained PtdChol Control 2h	Yes (0,236)	0,4206
		Sustained PtdChol Control 4h	Yes (0,809)	1,965
		Sustained PtdChol Ethanol 50 mM 2h	Yes (0,647)	1,893
		Sustained PtdChol Ethanol 50 mM 4h	No (0,005)	2,533
		Sustained PtdChol Ethanol 100 mM 2h	Yes (0,062)	1,976
		Sustained PtdChol Ethanol 100 mM 4h	Yes (0,249)	4,373
Figure 4	A	PtdSer Control, Ethanol 0 mM	Yes (0,570)	0,3209
		PtdSer Control, Ethanol 50 mM	Yes (0,668)	0,2035
		PtdSer Control, Ethanol 100 mM	Yes (0,493)	0,06095
		PtdSer Desipramine, Ethanol 0 mM	Yes (0,827)	0,251
		PtdSer Desipramine, Ethanol 50 mM	Yes (0,151)	0,337
		PtdSer Desipramine, Ethanol 100 mM	Yes (0,067)	0,1578
		PtdSer GW4869, Ethanol 0 mM	Yes (0,750)	0,2438
		PtdSer GW4869, Ethanol 50 mM	Yes (0,833)	0,2599
		PtdSer GW4869, Ethanol 100 mM	Yes (0,081)	0,1855
		PtdSer Cyclosporin A, Ethanol 0 mM	Yes (0,668)	0,2805
		PtdSer Cyclosporin A, Ethanol 50 mM	Yes (0,328)	0,1327
		PtdSer Cyclosporin A, Ethanol 100 mM	Yes (0,207)	0,1739
		PtdEtn Control, Ethanol 0 mM	Yes (0,540)	0,2044
		PtdEtn Control, Ethanol 50 mM	Yes (0,790)	0,1311
		PtdEtn Control, Ethanol 100 mM	Yes (0,742)	0,1562
		PtdEtn Desipramine, Ethanol 0 mM	Yes (0,882)	0,2719
		PtdEtn Desipramine, Ethanol 50 mM	Yes (0,232)	0,432
		PtdEtn Desipramine, Ethanol 100 mM	Yes (0,164)	0,2334
		PtdEtn GW4869, Ethanol 0 mM	Yes (0,572)	0,2006
		PtdEtn GW4869, Ethanol 50 mM	Yes (0,752)	0,162
		PtdEtn GW4869, Ethanol 100 mM	Yes (0,490)	0,271
		PtdEtn Cyclosporin A, Ethanol 0 mM	Yes (0,751)	0,1022
		PtdEtn Cyclosporin A, Ethanol 50 mM	Yes (0,776)	0,189
		PtdEtn Cyclosporin A, Ethanol 100 mM	Yes (0,305)	0,1697

		PtdChol Control, Ethanol 0 mM	Yes (0,396)	0,2719
		PtdChol Control, Ethanol 50 mM	Yes (0,435)	0,2802
		PtdChol Control, Ethanol 100 mM	Yes (0,835)	0,4207
		PtdChol Desipramine, Ethanol 0 mM	Yes (0,091)	0,4807
		PtdChol Desipramine, Ethanol 50 mM	Yes (0,372)	0,4952
		PtdChol Desipramine, Ethanol 100 mM	Yes (0,113)	0,2016
		PtdChol GW4869, Ethanol 0 mM	Yes (0,173)	0,04135
		PtdChol GW4869, Ethanol 50 mM	Yes (0,291)	0,2595
		PtdChol GW4869, Ethanol 100 mM	Yes (0,522)	0,215
		PtdChol Cyclosporin A, Ethanol 0 mM	Yes (0,680)	0,552
		PtdChol Cyclosporin A, Ethanol 50 mM	Yes (0,954)	0,2977
		PtdChol Cyclosporin A, Ethanol 100 mM	Yes (0,509)	0,1251
	B	Control	Yes (0,641)	389840000
		Ethanol	Yes (0,359)	1322923000
		Desipramine	No (0,014)	1090299000
		GW4869	Yes (0,110)	528909582
		Cyclosporin A	Yes (0,939)	199000000
